# Causative role of a novel intronic indel variant in *FBN1* and maternal germinal mosaicism in Marfan syndrome

**DOI:** 10.1186/s13023-024-03139-4

**Published:** 2024-05-21

**Authors:** Ying Bai, Yue Sun, Chenguang Yu, Yanjie Xia, Jing Wu, Li Wang, Yong Gao, Xin Tu, Xiangdong Kong

**Affiliations:** 1 Genetic and Prenatal Diagnosis Center, Department of Obstetrics and Gynecology, the First Affiliated Hospital of Zhengzhou University, Zhengzhou, 450052 China; 2 Key Laboratory of Molecular Biophysics of the Ministry of Education, College of Life Science and Technology and Center for Human Genome Research, Huazhong University of Science and Technology, Wuhan, 430074 China; 3 Department of Pediatrics, the First Affiliated Hospital of Zhengzhou University, Zhengzhou, 450052 China; 4 BGI-Wuhan, BGI-Shenzhen, Wuhan, 430074 China

**Keywords:** Marfan syndrome, *FBN1*, Cis variants, Intronic indel variant, Germinal mosaicism

## Abstract

**Background:**

Marfan syndrome (MFS) is an autosomal dominant connective tissue disease with wide clinical heterogeneity, and mainly caused by pathogenic variants in fibrillin-1 (*FBN1*).

**Methods:**

A Chinese 4-generation MFS pedigree with 16 family members was recruited and exome sequencing (ES) was performed in the proband. Transcript analysis (patient RNA and minigene assays) and *in silico* structural analysis were used to determine the pathogenicity of the variant. In addition, germline mosaicism in family member (Ι:1) was assessed using quantitative fluorescent polymerase chain reaction (QF-PCR) and short tandem repeat PCR (STR) analyses.

**Results:**

Two cis-compound benign intronic variants of *FBN1* (c.3464–4 A > G and c.3464-5G > A) were identified in the proband by ES. As a compound variant, c.3464-5_3464-4delGAinsAG was found to be pathogenic and co-segregated with MFS. RNA studies indicated that aberrant transcripts were found only in patients and mutant-type clones. The variant c.3464-5_3464-4delGAinsAG caused erroneous integration of a 3 bp sequence into intron 28 and resulted in the insertion of one amino acid in the protein sequence (p.Ile1154_Asp1155insAla). Structural analyses suggested that p.Ile1154_Asp1155insAla affected the protein’s secondary structure by interfering with one disulfide bond between Cys^1140^ and Cys^1153^ and causing the extension of an anti-parallel β sheet in the calcium-binding epidermal growth factor-like (cbEGF)13 domain. In addition, the asymptomatic family member Ι:1 was deduced to be a gonadal mosaic as assessed by inconsistent results of sequencing and STR analysis.

**Conclusions:**

To our knowledge, *FBN1* c.3464-5_3464-4delGAinsAG is the first identified pathogenic intronic indel variant affecting non-canonical splice sites in this gene. Our study reinforces the importance of assessing the pathogenic role of intronic variants at the mRNA level, with structural analysis, and the occurrence of mosaicism.

**Supplementary Information:**

The online version contains supplementary material available at 10.1186/s13023-024-03139-4.

## Introduction

Marfan syndrome (MFS; OMIM#154,700) is a relatively common (incidence of 2–3/10,000) autosomal dominant connective tissue disorder characterized by variable clinical features of the ocular, skeletal, and cardiovascular systems [1, 2]. Myopia and ectopia lentis are the most prominent ocular features of MFS and are present in approximately 60% of patients with this condition [2]. The most life-threatening aspect of MFS is an aortic root aneurysm with subsequent dissection and rupture. The wide variety of symptoms and their severity make MFS a disease difficult to diagnose. Molecular screening is not mandatory for MFS diagnosis; however, this approach would be very useful, especially in cases of patients with incomplete phenotypes.

Fibrillin-1 gene (*FBN1*), located on 15q21.1, has been identified as the major causative gene of MFS. *FBN1* includes 66 exons and encodes a 2871 aa structural macromolecule, fibrillin-1. Fibrillin-1 polymerizes into microfibrils and is present in all connective tissues, providing stability and elasticity to many tissues. This protein comprises 47 cysteine-rich epidermal growth factor (EGF)-like domains and seven transforming growth factor-β1 binding protein -like domains [3]. Variants in *FBN1* have been reported in a wide range of autosomal dominant heritable connective tissue disorders, including MFS [4], acromicric dysplasia (OMIM 102,370) [5], isolated ectopia lentis (OMIM 129,600) [6], geleophysic dysplasia type 2 (OMIM 614,185) [5], Marfan lipodystrophy syndrome (OMIM 616,914) [7], MASS syndrome (OMIM 604,308) [8], stiff skin syndrome (OMIM 184,900) [9], Weill–Marchesani syndrome type 2 (OMIM 608,328) [10], and familial ascending aortic aneurysm and aortic dissection [11].

Pathogenic variants of *FBN1* have been identified in more than 90% of patients with classic MFS syndrome. As of December 2022, 1847 variants of *FBN1* have been recorded in the Universal Mutation Database (http://www.umd.be/FBN1/-), 2792 likely pathogenic or pathogenic variants included in the ClinVar database (https://www.ncbi.nlm.nih.gov/clinvar/), and 3272 variants reported in the Human Gene Mutation Database (HGMD, https://www.hgmd.cf.ac.uk/ac/index.php). The variant spectrums of *FBN1* in the HGMD and ClinVar databases are composed of missense and nonsense variants (2022, 61.8%; 1358, 48.6%), small deletions and small insertions (711, 21.7%; 751, 26.9%), splice site variants (375, 11.5%; 218, 7.8%), and structural variants (116, 3.5%; 42, 1.5%). Reports of intronic indel variants affecting non-canonical splice sites in *FBN1* that cause MFS are rare. This may be associated with the less predictable information provided by the software, limited numbers of family members of patients, and need of further functional research. In routine molecular diagnosis, mosaicism is easily overlooked and obtaining proof of mosaicism is challenging. Although mosaicism in MFS is not common, it is of great significance for accurate diagnosis and genetic counseling.

In the present study, a novel *FBN1* intronic indel variant, c.3464-5_3464-4delGAinsAG, was identified in a 6-year-old boy with MFS through exome sequencing (ES) analysis, and co-segregated with bilateral lens dislocation, aortic sinus dilatation, and minor skeletal involvement. We tested the effect of the variant c.3464-5_3464-4delGAinsAG on gene splicing using RNA samples form patients and a minigene assay and evaluated the impact of potential protein structure changes using three-dimensional structure modeling. Additionally, we found that the family member Ι:1 displayed germline mosaicism for the *FBN1* c.3464-5_3464-4delGAinsAG variant and exhibited normal features in ocular and cardiovascular systems.

## Materials and methods

### Patients

The proband visited our hospital because of sudden vision impairment. The z-score of the aortic root was calculated according to body mass index-adjusted nomograms for the aortic root using www.parameterz.com [12]. The proband had a positive family history (Chinese family from Henan Province), and seven affected family members out of 16 were recruited. MFS was diagnosed based on the revised Ghent criteria [13].

### ES and bioinformatic analysis

Genomic DNA was extracted from the peripheral blood using a Lab-Aid Nucleic Acid Isolation Kit (Zeesan, Xiamen, China). ES was performed on the proband using Illumina library construction and capture kits (Illumina, San Diego, CA, United States) according to standard instructions, and paired-end reads with > 100× coverage were obtained using an Illumina Novaseq 6000 Sequencer (Illumina, San Diego, CA, United States). High-quality reads were mapped to the human reference genome, GRCh37/hg19. The Efficient Genosome Interpretation System (EGIS; Sierra Vast Bio-Medical, Shanghai, China) was used for mapping, variant calling, variant annotation, single-nucleotide variants and small indel (SNV/INDEL) analysis, and ES-based copy number variation analysis [14]. Exonic and splice site variants with a minor allele frequency (MAF) of less than 0.01 in dbSNP database, 1000 Genomes, Exome Aggregation Consortium and gnomAD database were selected for further analyses.

The pathogenicity of all variants was evaluated according to the latest guidelines of the American College of Medical Genetics and Genomics (ACMG) [15]. The pathogenicity of the identified intronic variants of *FBN1* was assessed in the EGIS using various tools, including SpliceAI, dbSNV_ADA, dbSNV_RF, mmsplice_delta_logit_psi, mmsplice_pathogenicity, Spidex-Zscore. Berkeley Drosophila Genome Project Searches (BDGP, https://www.fruitfly.org/seq_tools/splice.html) were used to predict the intronic variants, c.3464–4 A > G and c.3464-5_3464-4delGAinsAG. Sanger sequencing was performed to confirm the potential variants of *FBN1* using the primer pairs listed in Supplementary Table 1.

### Linkage analysis

Quantitative fluorescent polymerase chain reaction (QF-PCR) and short tandem repeat PCR (STR) analyses were performed to confirm the genetic relationship of the family members using the GoldeneyeTM DNA ID System 20 A Kit (Peoplespot, Beijing, China) and a polymorphic microsatellite marker, D15S992, linked to *FBN1*. The markers were genotyped using an ABI 3500 capillary DNA Sequencer (Applied Biosystems, Foster City, CA).

### Transcriptional study

Peripheral blood mononuclear cells (PBMCs) were isolated using Ficoll-Paque Plus (GE Healthcare) and TRIzol reagent (Invitrogen, Carlsbad, CA, United States). A minigene-splicing assay was performed to assess the effects of c.3464-5_3464-4delGAinsAG on RNA splicing. Briefly, a 4679 bp fragment of the *FBN1* gene, including exons 25–31 and introns 26–30, was amplified from the proband DNA’s using a KOD FX polymerase kit (Toyobo, Osaaka, Japan) with specific primers (p-IN25F and p-E31R) linking the EcoR1 and HindIII restriction enzyme sites (Supplementary Table 1). The fragment was isolated and inserted into the pcDNA3.1 (-) vector using the ClonExpress® II One Step Cloning kit (Vazyme Biotech Co., Ltd, Nanjing, China). Finally, the minigene constructions were sequenced to check for the presence of the wild-type or mutant allele after plasmid extraction (Omega, Doraville, GA, United States).

One day before transfection, human embryonal kidney (HEK) 293T cells were seeded on a 12-well plate, grown to 80% confluence, and transfected with 1 µg plasmid DNA in the group of minigenes using Lipofectamine 2000 (Invitrogen, Carlsbad, CA, United States) according to the manufacturer’s instructions. Twenty-four hours after transfection, cells were harvested using the TRIzol reagent (Invitrogen, Carlsbad, CA, United States).

RNA was isolated from PBMCs and HEK293T cells using the standard TRIzol (Invitrogen) procedure and reverse-transcribed into complementary DNA (cDNA) using the HiScript II 1st Strand cDNA Synthesis kit (Vazyme Biotech Co.,Ltd., Nan jing, China). A 605 bp fragment of *FBN1* cDNA was amplified using primers RT-26 F and RT-31R (Supplementary Table 1). PCR products were resolved in 1.5% agarose gel electrophoresis and sequenced on an ABI 3500 capillary DNA sequencer (Applied Biosystems, Foster City, CA).

### The three-dimensional structure modeling

The calcium-binding epidermal growth factor-like (cbEGF) 13–14 domain of human fibrillin-1 was modeled as follows. We obtained a template (PDB entry 1LMJ) for modeling using the Protein Data Bank (PBD, http://www.rcsb.org) [16]. The wild-type cbEGF13-14 domain of fibrillin-1 (cbEGF13-14^WT^, amino acids:1113–1196) was modeled using the SWISS-PDB Viewer (https://swissmodel.expasy.org/) and then visualized in Discovery Studio (DS) 2016 (Dassault Systèmes, Vélizy-Villacoublay, France). The best quality model was selected based on qualitative model energy analysis scores and validated using the SAVES v6.0 (https://saves.mbi.ucla.edu/) and ProSAweb (https://prosa.services.came.sbg.ac.at/prosa.php) servers. The mutant cbEGF13-14 model (cbEGF13-14^M^, p.Ile1154_Asp1155insAla) was generated by inputting the corresponding mutant amino acid sequences using the same analytical process described above.

## Results

### Pedigree and clinical features

**Fig. 1:**
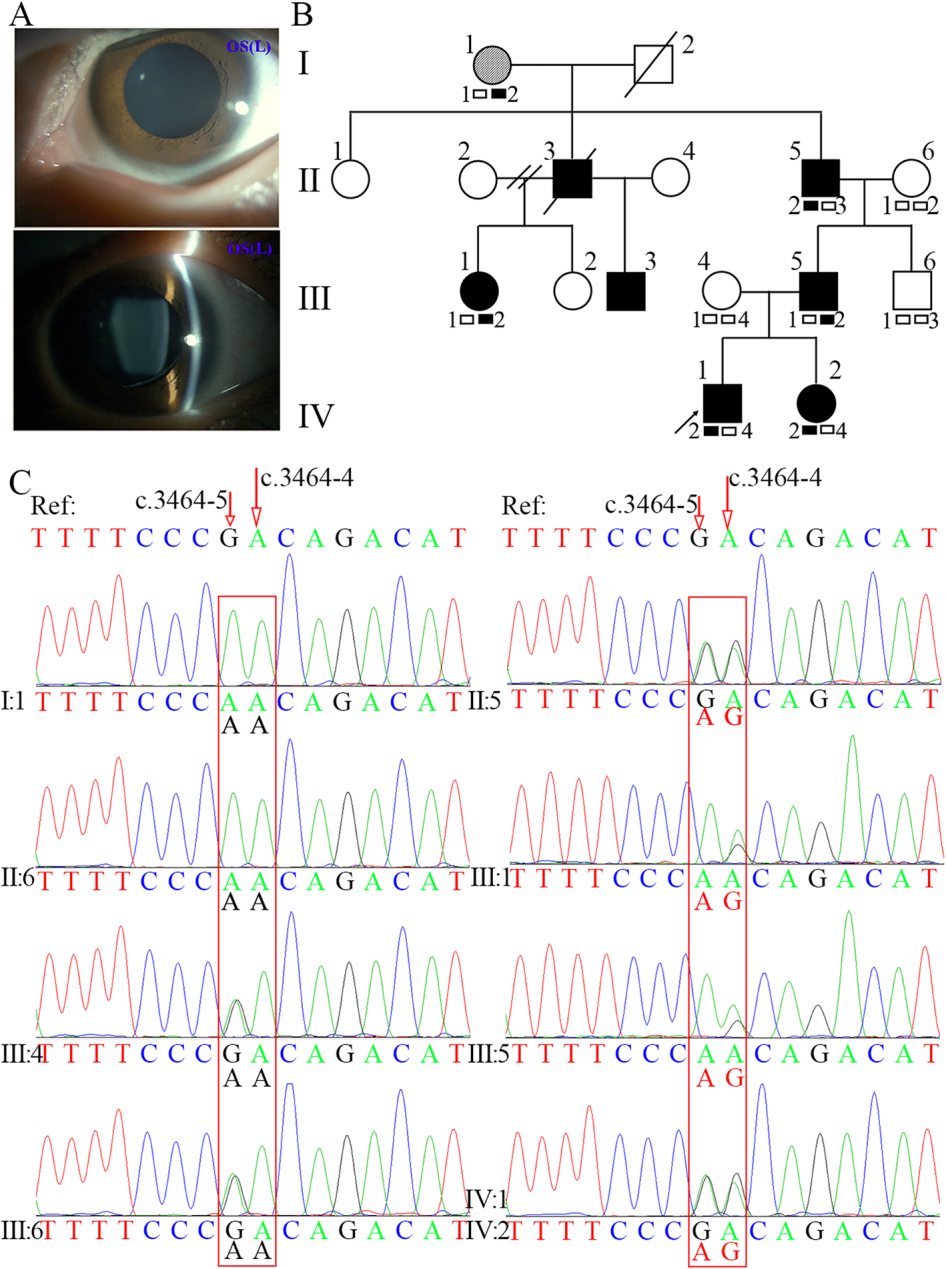
Images, pedigree and genetic information of the MFS family. **A** Ectopia lentis of the proband (IV:1). Images were taken by slit lamp after pupil dilation. **B** Pedigree of four generation family with MFS. Squares indicate males and circles indicate females. Patients diagnosed with MFS are in black below. Arrow marks the proband. I, II, III, IV refer to the first, second, third, and fourth generations of the family, respectively. A diagonal line indicates that a subject is deceased. The mosaicism is indicated by hatched symbol. Linkage analysis was performed with one polymorphic microsatellite marker (D15S992) linked to the *FBN1* gene. Genotypic results are exhibited under each symbol. The allele 2 co-segregates with affected individuals expect member Ι:1, suggesting that the disease-causing gene in the family is linked to *FBN1.* Note that member Ι:1 had two affected sons. These results implied that member Ι:1 is a germline mosaicism of c.3464-5_3464-4delGAinsAG. **C** The PCR and direct sequencing of the genomic DNA in the family members. The red boxes indicate the sequence variant. People with mutant allele AG in c.3464-5_3464-4 of *FBN1* gene are in red

The proband IV:1 was a 6-year-old Chinese boy (height, 124 cm; weight, 23.3 kg). One month prior to admission, the patient had decreased visual acuity [*oculus dexter* (OD): 0.4, *oculus sinister* (OS): 0.06] and was admitted to our hospital for treatment. An ophthalmic examination revealed decreased visual acuity, bilateral lens dislocation (Fig. 1A), and normal intraocular pressure (OD: 13 mmHg, OS: 14 mmHg). His left eye vision was restored after pars plana vitrectomy and scleral intraocular lens fixation surgery (OS: 0.5). Cardiac Doppler ultrasound suggested that the proband had aortic sinus dilatation (aortic sinus diameter: 28 mm, Z-score: 3.63), an atrial septal defect, and mild tricuspid regurgitation. The pedigree of the four-generation MFS family is shown in Fig. 1B, and detailed clinical information is shown in Table 1. Seven of the 16 family members that were tested presented with MFS. All affected members of this family manifested similar clinical symptoms, mainly in the ocular and cardiovascular systems. All patients presented bilateral lens dislocation in the ocular system. Cardiovascular system abnormalities were observed in patients II:3, II:5, III:5, and IV:1. Patient II:3 died of sudden cardiac death at the age of 30. Echocardiography in patient II:5 showed dilatations of the ascending aorta (42 mm), left atrial and ventricular enlargement (left atrial diameter: 42 mm and left ventricular diameter: 57 mm), and moderate aortic valve, mild mitral, and tricuspid insufficiencies. Patient III:5 had mitral valve replacement at the age 27 years (echocardiography data not available). The main skeletal system abnormalities were pectus carinatum and plain pes planus.

**Table 1:** Clinical detail of the family members

Family members	Genotype information (FBN1)	type	Gender	Age (years)	Height (cm)	Ectopia lentis(R/L)	Aortic dilation	other
I:1	c.3464-5G > A	hom	F	90	170	-/-	NA	-
II:3	NA	NA	M	-	180	+/+	+	due to cardiovascular accident died at 30 years of age
II:5	**c.3464-5_3464-4delGAinsAG**	het	M	56	174	+/+	+	ascending aorta dilatation, left-ventricular dilatation aortic insufficiency, mild mitral and tricuspid insufficiency; Pectus carinatum deformit, plain pes planus
II:6	c.3464-5G > A	hom	F	58	160	-/-	-	-
III:1	**c.3464-5_3464-4delGAinsAG**/c.3464-5G > A	het	F	45	171	+/+	NA	NA
III:3	NA	NA	M	40	169	+/+	NA	NA
III:4	c.3464-5G > A	het	F	32	168	-/-	-	-
III:5	**c.3464-5_3464-4delGAinsAG**/c.3464-5G > A	het	M	33	173	+/+	+	decreased visual acuity, undergo mitral valve replacement at 27 years of age; Myopia > 3 diopters; Pectus carinatum deformit, plain pes planus
III:6	c.3464-5G > A	het	M	31	172	-/-	-	-
IV:1	**c.3464-5_3464-4delGAinsAG**	het	M	6	124	+/+	+	decreased visual acuity, atrial septal defect, aortic sinus dilatation, mild tricuspid regurgitation; Pectus carinatum deformit, plain pes planus
IV:2	**c.3464-5_3464-4delGAinsAG**	het	F	3	NA	+/+	-	mild mitral/tricuspid regurgitation

*F* female, *M* male, *L* left, *R* right, *NA* not available, *WT* wild type. The mutant allele was indicated with bold

### Analysis of exome sequencing

After filtering (MAF < 0.01), three variants in the exon and splice regions of *FBN1* (c.3605G > T, c.3606 C > T, and c.3464–4 A > G) were identified using ES of the proband. However, each variant was predicted to be either a variant of unknown significance (VUS) or benign (Supplementary Table 2). We then focused on all variants of *FBN1* and found that each pair of two adjacent variants (c.3605G > T and c.3606 C > T; c.3464–4 A > G and c.3464-5G > A) was in cis (Supplementary Figs. 1 and 2). Two indel variants, c.3605_3606delGCinsTT p. (Ser1202Ile) and c.3464-5_3464-4delGAinsAG (Table 2), were not found in the dbSNP database, 1000 Genomes, Exome Aggregation Consortium and gnomAD database. Sanger sequencing revealed that c.3605_3606delGCinsTT p. (Ser1202Ile) and c.3464-5_3464-4delGAinsAG were inherited from the mother (III:4, Supplementary Fig. 3) and father (III:5, Fig. 1), respectively. The probands mother (III:4) did not have any clinical signal of Marfan syndrome, and therefore the pathogenicity of c.3605_3606delGCinsTT was discarded. The c.3464-5_3464-4delGAinsAG variant was found in all tested patients and was heterozygous in patients II:5, III:1, III:5, IV:1, and IV:2, and absent in all unaffected members (Ι:1, II:6, III:4 and III:6). Prediction of c.3464-5_3464-4delGAinsAG revealed potential abnormal splicing resultant from the introduction of a single alanine insertion, p.Ile1154_Asp1155insAla, using BDGP (Supplementary Fig. 4). According to the ACMG guidelines, the c.3464-5_3464-4delGAinsAG variant is classified as “likely pathogenic (PP1_Strong + PM2_supporting + PP3 + PP4).”

**Table 2:** The cis variants in *FBN1* gene

Gene	transcript	Exon	hgvs.c	hgvs.p	type	Genotype	dbsnp	ExAC_EAS	ref/alt	inherit	Variant type (ACMG)
***FBN1***	**NM_000138.4**	**intron28**	**c.3464-5_3464-4delGAinsAG**	**-**	**splice_region_variant**	**het**	**-**	**-**	**51/45**	**Father**	**Novel/LP (PP1_Strong+** **PM2_supporting + PP3 + PP4)**
*FBN1*	NM_000138.4	exon30	c.3605_3606delGCinsTT	p.Ser1202Ile	Missense_variant	het	-	-	29/23	mother	VUS (BS2 + PM2_supporting)

Family member II:5 carried the heterozygous variant c.3464-5_3464-4delGAinsAG (GA/AG), but his mother, Ι:1, had the homozygous variant c.3464-5G > A (AA/AA). The paternal relationship was confirmed by QF-PCR (Supplementary Fig. 5) and linkage analysis of one high polymorphic microsatellite marker, D15S992 (Fig. 1B, Supplementary Fig. 6). The capillary electrophoresis results showed two products (234 bp and 254 bp) in member Ι:1, with the mutant peaking at 254 bp. Based on these results and those of two affected sons (patients II:3 and II:5), we speculated that family member Ι:1 had germline mosaicism of c.3464-5_3464-4delGAinsAG.

### Aberrant splicing for the c.3464-5_3464-4delGAinsAG variant

**Fig. 2:**
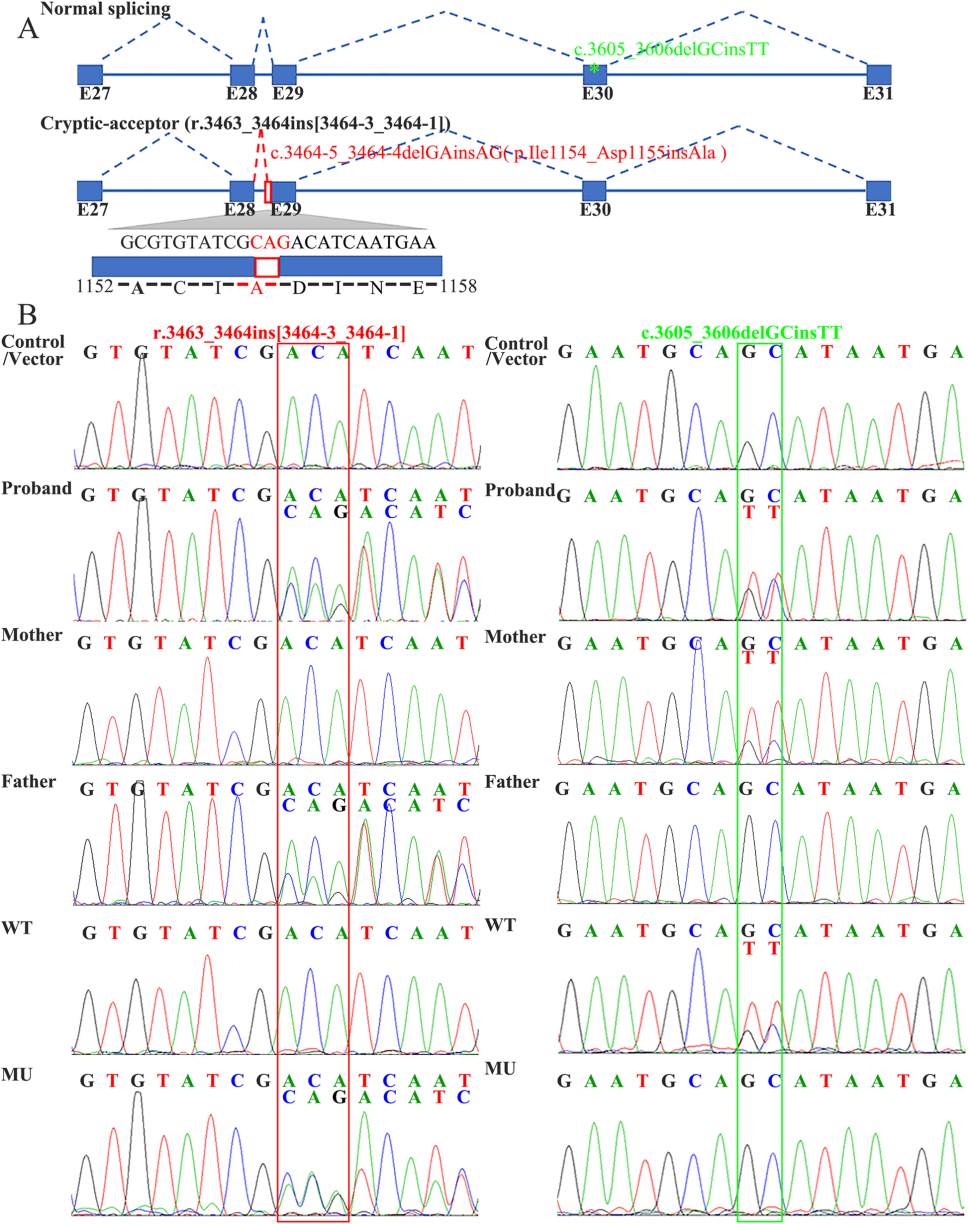
Transcript analyses of *FBN1* variant c.3464-5_3464-4delGAinsAG. **A** Schematic of detected missplicing for the patients. The variant c.3464-5_3464-4delGAinsAG (red asterisk) with c.3605_3606delGCinsTT (green asterisk) in trans. **B** Sanger sequencing of the RT-PCR products in patients and minigene assays. Compared with the control and wild-type clones, c.3464-5_3464-4delGAinsAG induces the insertion of 3 bp sequence (CAG, red rectangle) in intron 28. WT: wild-type, MU: mutant-type

*FBN1* mRNA studies using blood-derived cDNA from patients and minigene assays established that the intronic variant *FBN1* c.3464-5_3464-4delGAinsAG induces an in-frame splicing defect (3 bp retention in intron 28), namely, the use of a cryptic-acceptor splice site in intron 28 (r.3463_3464ins [3464-3_3464-1], p.Ile1154_Asp1155insAla; Fig. 2A, C). No significant difference was observed among RNA samples of patients and controls or mutant and wild-type clones analyzed using agarose gel electrophoresis (Supplementary Fig. 7). However, *FBN1* mRNA studies showed mis-splicing events in the proband (IV:1) and his father (III:5) and mutant-type clones absent in the proband’s mother (III:4), control, and wild-type clones. Notably, minigene constructions were generated with the proband’s DNA with a paternal pathogenic variant (c.3464-5_3464-4delGAinsAG) and a maternal benign variant (c.3605_3606delGCinsTT) in trans orientation (Fig. 2A, Supplemental Fig. 3). Sanger sequencing of the RT-PCR products of the minigene assay revealed a correctly spliced *FBN1* transcript encompassing the benign variant c.3605_3606delGCinsTT in wild-type cells, and a mis-spliced *FBN1* transcript in mutant-type cells without the benign variant c.3605_3606delGCinsTT (Fig. 2B).

### Structural analysis of p.Ile1154_Asp1155insAla

**Fig. 3:**
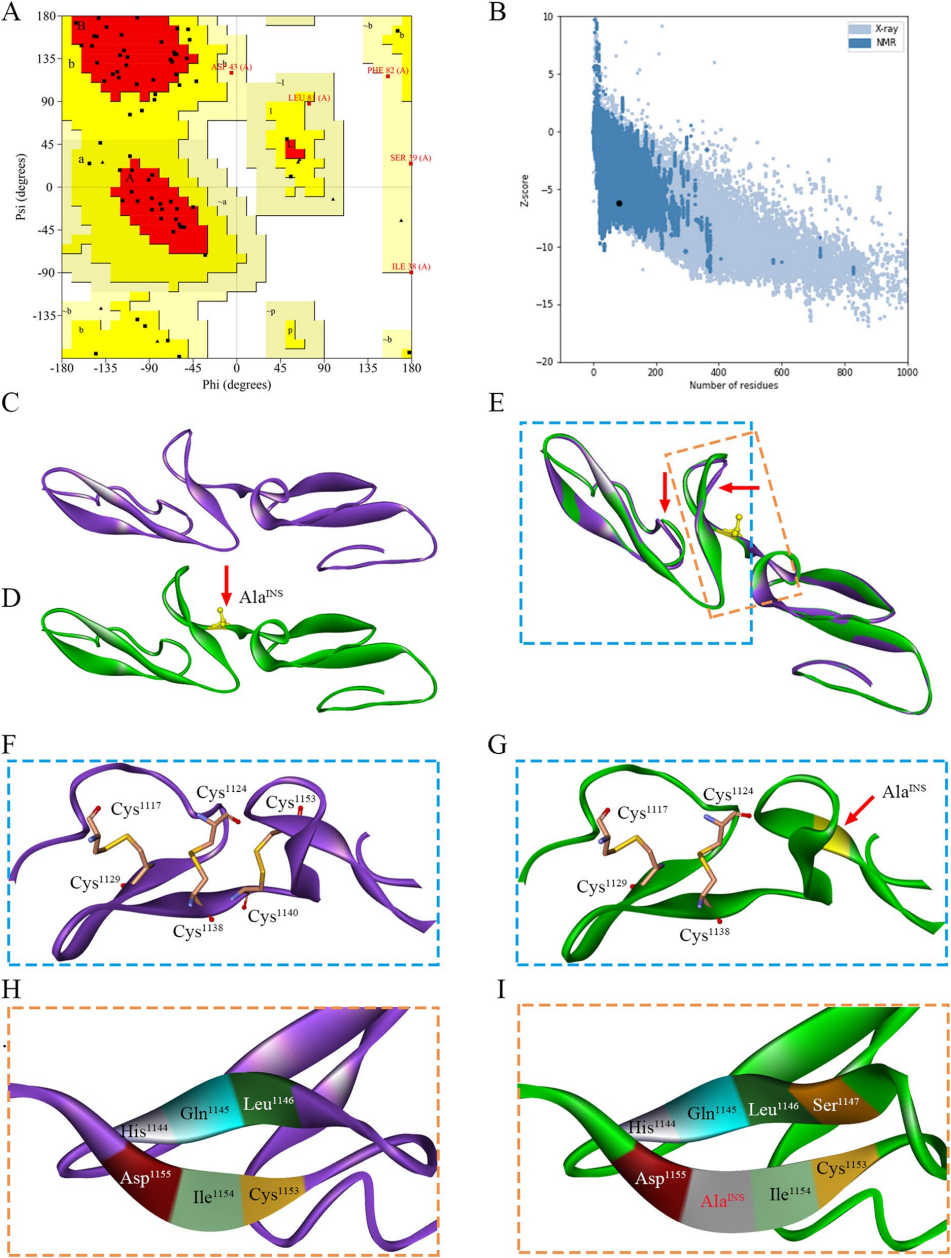
The 3D structural and molecular interaction analyses of the wild-type (cbEGF13-14^WT^) and mutant-type cbEGF13-14 (cbEGF13-14^M^) domains of fibrillin-1. Residues distribution in the correspondent regions of Ramachandran plot (**A**) and z-score validation (**B**) of the homology model of cbEGF13-14 domain of fibrillin-1. Homology model of cbEGF13-14^WT^ (**C**) and cbEGF13-14^M^ (**D**) of fibrillin-1. The inserted amino acid is indicated in a red arrow and marked yellow. The superimposition portrayal of cbEGF13-14 ^WT/M^ models. The red arrows indicated the predicted mild structural differences (**E**). Structural and molecular analyses expose that insertion alanine (Ala^INS^) in the junction of cbEGF13-14 domain alters the secondary structure as a result of broken disulfide bond between Cys^1140^ and Cys^1153^ in cbEGF13 (**F**–**G**) and extension of an anti-parallel β sheet (**H**-**I**). Disulfide bonds of cbEGF13 are depicted by yellow sticks and marked in a blue box. Comparison to disulfide bonds (Cys^1117^-Cys^1129^, Cys^1124^-Cys^1138^ and Cys^1153^-Cys^1140^) in cbEGF13-14^WT^ (**F**), one disulfide bond (Cys^1153^-Cys^1140^) was destroyed in the cbEGF13-14^M^ (**G**). The anti-parallel β sheet of the C-terminal of cbEGF13 is marked with an orange box. The main interactions of β sheet involve the side chains of 6 amino acids (His^1114^, Gln^1145^, Leu^1146^, Cys^1153^, Ile^1154^, Asp^1155^) in the C-terminal of cbEGF13^WT^(**H**), but 8 amino acids (His^1114^, Gln^1145^, Leu^1146^, Ser^1147^, Cys^1153^, Ile^1154^, Ala^INS^, Asp^1155^) in cbEGF13 ^M^(**I**)

The p.Ile1154_Asp1155insAla variant is located at the junction of the cbEGF13 and cbEGF14 domains in human fibrillin-1. To determine whether the p.Ile1154_Asp1155insAla variant affected the cbEGF13 and/or cbEGF14 domains, we derived three-dimensional models of the cbEGF13-14 domains. PDB entry 1LMJ (40.48% similarity with cbEGF13-14) was selected as the template for modeling. Stereochemical validation revealed that approximately 92.6% of the residues occupied the most favored region and an additional allowed region in the Ramachandran plot (Fig. 3A). The Z-score (-6.18) analysis using the ProSAweb server validated that the quality of the cbEGF13-14domain model was sufficient for further analysis (Fig. 3B). Graphical superimposition of cbEGF13-14^WT^ and cbEGF13-14^M^ revealed minor local deviations around the insertion of mutant position (Fig. 3C-E). Based on the comparison analysis between models of cbEGF13-14^WT^ and cbEGF13-14^M^, it was likely that the insertion of the alanine residue (Ala^INS^) in the junction of cbEGF13-14 altered the secondary structure, which disrupted the formation of the C5–C6 disulfide bond between Cys^1140^ and Cys^1153^ in cbEGF13 ^M^ (Fig. 3F, G), and generated the extension of an anti-parallel β sheet with Ala^INS^ and Ser^1147^ in the C-terminal of cbEGF13^M^ (Fig. 3H, I). These molecular changes have been suggested to promote susceptibility to proteolysis [17].

## Discussion

In the present study, we performed clinical and molecular evaluations of seven members of a family of Chinese MFS patients exhibiting the phenotype of bilateral lens dislocation, aortic sinus dilatation and minor skeletal involvement, and found a novel intronic indel variant in a non-canonical splice site, c.3464-5_3464-4delGAinsAG. The two *FBN1* intronic variants, c.3464–4 A > G and c.3464-5G > A, were ignored during our initial ES analysis of the proband because c.3464–4 A > G was predicted to be benign by several prediction software (Supplementary Table 3) and c.3464-5G > A with MAF 0.35 was not present in EGIS (Supplementary Table 2). After determining the clinical features of the family members with MFS, all *FBN1* variants were loaded and reanalyzed. These two cis -intronic variants, c.3464–4 A > G and c.3464-5G > A, could be regarded as a combined indel variant c.3464-5_3464-4delGAinsAG. The pathogenicity of the c.3464-5_3464-4delGAinsAG variant was confirmed by co-segregation, transcription, and in silico analyses. Only 10 intronic indel variants affecting non-canonical splice sites have been included in ClinVar database and classified as likely benign [5] or VUS [5] (Supplemental Table 4). To our knowledge, this is the first report of a pathogenic intronic indel variant affecting non-canonical splice sites in *FBN1*.

Considering the limitations of bioinformatics tools for appropriate ‘indel’ annotation, the Human Genome Variation Society recommends that two variants separated by one or more nucleotides be described individually and not as a combined ‘indel’ (insertion-deletion) [18, 19]. These two different nomenclatures, as two separate events affecting the same allele or as a compound indel variant, may result in confusion regarding the reporting of variants and the significance of these variants among clinicians. Zo et al. reported that two cis variants in *IDH2*, Ile168Val and Arg172Gly, might be interpreted as “VUS” as a combined ‘indel’, but Arg172 is a well-known oncogenic hotspot that can be therapeutically actionable. However, this nomenclature may obscure clinically relevant and potentially actionable information [18, 20]. In contrast, the presence of both c.3464–4 A > G and c.3464-5G > A in *FBN1*, the benign variants constituted a compound indel variant, c.3464*-*5_3464-4delGAinsAG, and was associated with MFS. Zhang et al. [21] observed the cis-compound variants of Met822Thr and Arg1420Trp in *MYH7* in a large Chinese pedigree with hypertrophic cardiomyopathy, two variants that are relatively benign. Therefore, caution should be taken when interpreting two or more variants in a disease-causing gene with explicit association with clinical features of patients; one should (1) review the pileup for the orientation of the variants (cis vs. trans), (2) analyze the genotype − phenotype correlation in the family, (3) and determine the variant pathogenicity at the RNA or protein level.

Most *FBN1* pathogenic variants in cbEGF domain are predicted to cause disease by preventing the formation of disulfide bonds, altering functional calcium-binding affinity, significantly enhancing protease susceptibility, impairing folding of the cbEGF domain, affecting protein trafficking, and disrupting heparin binding [22]. Each cbEGF domain is stabilized by three disulfide bridges between C1–C3, C2–C4, and C5–C6 [23]. Formation of proper C2–C4 and C5–C6 bonds in the preceding EGF-like domain is critical for folding [24]. The p.C750G and p.C1320S cysteine pathogenic variants are associated with classical MFS, affecting C5 in cbEGF7 and C6 in cbEGF17 [17, 25]. Another cysteine pathogenic variant, p.C1182S, leads to neonatal MFS, which affects C5 in cbEGF14 [26]. These three pathogenic variants disrupt the formation of the C5–C6 disulfide bond, which induces short/long-range structural changes and increases proteolytic susceptibility. In addition, functional analyses of a fibrillin-1 fragment containing the p.C1182S pathogenic variant revealed reduced heparin binding, which may be associated with disease severity. Using steered molecular dynamics simulations, Haller et al. found that the C5–C6 bond stabilized calcium-dependent cbEGF-cbEGF inter-domain interfaces, and loss of the C5–C6 bond did not directly affect calcium binding [27]. Our results on the structural modeling analysis were in accordance with these findings. p.Ile1154_Asp1155insAla likely prevents the formation of the C5–C6 disulfide bond and influences the minor β-sheet of cbEGF13, which may further alter the hydrophobic interdomain interactions and increase proteolytic susceptibility. Although we did not investigate its effect further, it is still important to perform further functional studies on the molecular effects of this indel variant in vivo or in vitro.

The genotype/phenotype correlations of MFS have been recognized as an important consideration for diagnosis, precision medicine for prognosis, and clinical decisions [28, 29]. Although the associations between genotypes and phenotypes are not absolute, several associations have received widespread acceptance. The first is the increased risk for ectopia lentis, which is a characteristic feature of MFS. Ectopia lentis is more frequent in patients harboring cysteine-affecting disulfide bond variants than any other class of pathogenic variants [22, 30–32]. The present study also found similar results in all patients presenting with ectopia lentis and a novel pathogenic variant affecting the C5–6 disulfide bond. Additionally, most pathogenic variants in exons 24–32 are associated with neonatal MFS and other severe subtypes of MFS [6, 33]. Stheneur et al. [33] analyzed the prognostic features of patients with *FBN1* pathogenic variants in exons 24–32, confirmed this association, and narrowed it down to exons 25–26. In their study, 82% of patients with a pathogenic variant in exons 25–26 died before the age of one year. However, variants in this region have also been identified in patients with atypically severe and classic MFS [34]. In addition, a strong association between truncating pathogenic variants and cardiovascular defects in patients with MFS have been observed [35, 36]. Notably, there is wide phenotypic variability among family members carrying the same pathogenic variants. Bilateral lens dislocation occurred in the proband (IV:1) at age 6 due to decreased visual acuity; however, it occurred at age 33 in his father (III:5) and at age 30 in his grandfather (II:5). Li et al. reported that three different phenotypes were found in 12 family members with the same *FBN1* pathogenic variant (p.R545C), nine patients with ectopia lentis, one with aortic dissection, and one unaffected [37]. Further studies are needed to investigate whether other genes or factors are responsible for these differences.

Proving germinal mosaicism is challenging, especially maternal gonadal mosaicism in one parent. Although only blood was analyzed in the asymptomatic family member (I:1) in our study, no peak of the mutant allele was found by Sanger sequencing; maternal mosaicism was deduced from the recurrence of MFS in the two siblings. An estimated rate of 3.75% in parental mosaicism and 0.3% rate in affected patients were estimated in 1085 patients with thoracic aortic aneurysm and dissection (TAAD) by Yang et al. [38], and 4.8% parental mosaicism rate in 333 patients with *FBN1* pathogenic variants by Chesneau et al. [39]. Current mosaicism studies in *FBN1* show that parental mosaicism in MFS is not rare. The phenotypic features of *FBN1* mosaicism are usually mild or asymptomatic, and detailed evaluations are performed following the diagnosis of a family member with MFS [40]. A previous study by Arnaud et al. described five mosaic probands presenting with classical MFS features [41]. The effects of mosaic variants on individual phenotypes range from negligible to catastrophic. However, it is of great significance to detect parental mosaicism in clinical molecular diagnoses for genetic counseling, as it has a major impact on recurrence risk.

## Conclusions

In summary, the results of the present study reinforce the importance of enhanced analysis of the two cis intronic variants (c.3464–4 A > G and c.3464-5G > A) of *FBN1*, identified in a Chinese MFS family. According to our co-segregation results and functional studies, c.3464-5_3464-4delGAinsAG is pathogenic and is the first intronic indel variant affecting non-canonical splice sites in *FBN1.* Caution is recommended when analyzing two cis-variants of genes associated with clinical phenotypes, especially in patients with a family history of multiple diseases. Moreover, the present study enriches the spectrum of *FBN1* pathogenic variants and the genotype–phenotype correlation of this disease. Interestingly, unsuspected germinal mosaicism was inferred in an asymptomatic family member (I:1), suggesting that it is not a rare phenomenon in MFS, with a high proportion of sporadic cases. Germinal mosaicism should be considered because of its major impact on the evaluation of recurrence risk.

### Supplementary Information

**Supplementary Material 1.**

## Data Availability

The datasets used and analyzed during the current study are included in the article/Supplementary Material, further inquiries can be available from the corresponding author upon reasonable request.

## Abbreviations

MFS: Marfan syndrome
*FBN1*: Fibrillin-1
ES: Exome sequencing
QF-PCR: Quantitative fluorescent polymerase chain reaction
STR: Short tandem repeat
cbEGF: Calcium-binding epidermal growth factor-like
EGF: Epidermal growth factor
HGMD: Human Gene Mutation Database
EGIS: the Efficient Genosome Interpretation System
SNV/INDEL: Single-nucleotide variants and small insertion-deletion
ACMG: the American College of Medical Genetics and Genomics
BDGP: Berkeley Drosophila Genome Project Searches
PBMCs: Peripheral blood mononuclear cells
cDNA: Complementary DNA
PBD: Protein Data Bank
MAF: Minor allele frequency
OD: Oculus dexter
OS: Oculus sinister
VUS: Unknown significance
TAAD: Thoracic aortic aneurysm and dissection

## References

[1] Judge DP, Dietz HC (2005). Marfan’s syndrome. Lancet.

[2] Sakai LY, Keene DR, Renard M, De Backer J (2016). FBN1: the disease-causing gene for Marfan syndrome and other genetic disorders. Gene.

[3] Ramirez F, Dietz HC (2007). Fibrillin-rich microfibrils: structural determinants of morphogenetic and homeostatic events. J Cell Physiol.

[4] Dietz HC, Cutting GR, Pyeritz RE (1991). Marfan syndrome caused by a recurrent de novo missense mutation in the fibrillin gene. Nature.

[5] Le Goff C, Mahaut C, Wang LW (2011). Mutations in the TGFbeta binding-protein-like domain 5 of FBN1 are responsible for acromicric and geleophysic dysplasias. Am J Hum Genet.

[6] Robinson PN, Booms P, Katzke S (2002). Mutations of FBN1 and genotype-phenotype correlations in Marfan syndrome and related fibrillinopathies. Hum Mutat.

[7] Graul-Neumann LM, Kienitz T, Robinson PN (2010). Marfan syndrome with neonatal progeroid syndrome-like lipodystrophy associated with a novel frameshift mutation at the 3’ terminus of the FBN1-gene. Am J Med GEenet A.

[8] Dietz HC, McIntosh I, Sakai LY (1993). Four novel FBN1 mutations: significance for mutant transcript level and EGF-like domain calcium binding in the pathogenesis of Marfan syndrome. Genomics.

[9] Loeys BL, Gerber EE, Riegert-Johnson D (2010). Mutations in fibrillin-1 cause congenital scleroderma: stiff skin syndrome. Sci Transl Med.

[10] Faivre L, Gorlin RJ, Wirtz MK (2003). In frame fibrillin-1 gene deletion in autosomal dominant Weill-Marchesani syndrome. J Med Genet.

[11] Francke U, Berg MA, Tynan K (1995). A Gly1127Ser mutation in an EGF-like domain of the fibrillin-1 gene is a risk factor for ascending aortic aneurysm and dissection. Am J Hum Genet.

[12] Dallaire F, Bigras JL, Prsa M, Dahdah N (2015). Bias related to body mass index in pediatric echocardiographic Z scores. Pediatr Cardiol.

[13] Loeys BL, Dietz HC, Braverman AC (2010). The revised Ghent nosology for the Marfan syndrome. J Med Genet.

[14] Bai Y, Liu J, Xu J (2022). Long-read sequencing revealed extragenic and intragenic duplications of exons 56–61 in DMD in an asymptomatic male and a DMD patient. Front Genet.

[15] Richards S, Aziz N, Bale S (2015). Standards and guidelines for the interpretation of sequence variants: a joint consensus recommendation of the American College of Medical Genetics and Genomics and the Association for Molecular Pathology. Genet Med.

[16] Smallridge RS, Whiteman P, Werner JM, Campbell ID, Handford PA, Downing AK (2003). Solution structure and dynamics of a calcium binding epidermal growth factor-like domain pair from the neonatal region of human fibrillin-1. J Biol Chem.

[17] Booms P, Tiecke F, Rosenberg T, Hagemeier C, Robinson PN (2000). Differential effect of FBN1 mutations on in vitro proteolysis of recombinant fibrillin-1 fragments. Hum Genet.

[18] Lo YC, Narayan R, Nardi V, Lennerz JK (2021). Two in Cis variants-two worlds apart. Oncologist.

[19] Deans ZC, Fairley JA, den Dunnen JT, Clark C (2016). HGVS nomenclature in practice: an Example from the United Kingdom National External Quality Assessment Scheme. Hum Mutat.

[20] Stein EM, DiNardo CD, Pollyea DA (2017). Enasidenib in mutant IDH2 relapsed or refractory acute myeloid leukemia. Blood.

[21] Zhang M, Sun X, Wu G (2022). Effect of Cis-compound variants in MYH7 on hypertrophic cardiomyopathy with a mild phenotype. Am J Cardiol.

[22] Zeyer KA, Reinhardt DP (2015). Engineered mutations in fibrillin-1 leading to Marfan syndrome act at the protein, cellular and organismal levels. Mutat Res-Rev Mutat.

[23] Downing AK, Knott V, Werner JM, Cardy CM, Campbell ID, Handford PA (1996). Solution structure of a pair of calcium-binding epidermal growth factor-like domains: implications for the Marfan syndrome and other genetic disorders. Cell.

[24] Reinhardt DP, Ono RN, Sakai LY (1997). Calcium stabilizes fibrillin-1 against proteolytic degradation. J Biol Chem.

[25] Vollbrandt T, Tiedemann K, El-Hallous E (2004). Consequences of cysteine mutations in calcium-binding epidermal growth factor modules of fibrillin-1. J Biol Chem.

[26] Kirschner R, Hubmacher D, Iyengar G (2011). Classical and neonatal Marfan syndrome mutations in fibrillin-1 cause differential protease susceptibilities and protein function. J Biol Chem.

[27] Haller SJ, Roitberg AE, Dudley AT (2020). Steered molecular dynamic simulations reveal Marfan syndrome mutations disrupt fibrillin-1 cbEGF domain mechanosensitive calcium binding. Sci Rep-UK.

[28] Landis BJ, Veldtman GR, Ware SM (2017). Genotype-phenotype correlations in Marfan syndrome. Heart.

[29] Wang Y, Li X, Li R, Yang Y, Du J (2018). Identification of novel causal FBN1 mutations in pedigrees of Marfan syndrome. Int J Genomics.

[30] Schrijver I, Liu W, Brenn T, Furthmayr H, Francke U (1999). Cysteine substitutions in epidermal growth factor-like domains of fibrillin-1: distinct effects on biochemical and clinical phenotypes. Am J Hum Genet.

[31] Faivre L, Collod-Beroud G, Loeys BL (2007). Effect of mutation type and location on clinical outcome in 1,013 probands with Marfan syndrome or related phenotypes and FBN1 mutations: an international study. Am J Hum Genet.

[32] Martinez-Quintana E, Rodriguez-Gonzalez F, Garay-Sanchez P, Tugores A (2014). A novel fibrillin 1 gene mutation leading to marfan syndrome with minimal cardiac features. Mol Syndromol.

[33] Stheneur C, Faivre L, Collod-Beroud G (2011). Prognosis factors in probands with an FBN1 mutation diagnosed before the age of 1 year. Pediatr Res.

[34] Maeda J, Kosaki K, Shiono J, Kouno K, Aeba R, Yamagishi H (2016). Variable severity of cardiovascular phenotypes in patients with an early-onset form of Marfan syndrome harboring FBN1 mutations in exons 24–32. Heart Vessels.

[35] Baudhuin LM, Kotzer KE, Lagerstedt SA (2015). Increased frequency of FBN1 truncating and splicing variants in Marfan syndrome patients with aortic events. Genet Med.

[36] Wang WJ, Han P, Zheng J (2013). Exon 47 skipping of fibrillin-1 leads preferentially to cardiovascular defects in patients with thoracic aortic aneurysms and dissections. J Mol Med.

[37] Li Y, Xu J, Chen M (2016). A FBN1 mutation association with different phenotypes of Marfan syndrome in a Chinese family. Clin Chim Acta.

[38] Yang H, Zhu G, Zhou W (2022). A systematic study of mosaicism in heritable thoracic aortic aneurysm and dissection. Genomics.

[39] Chesneau B, Plancke A, Rolland G (2021). Parental mosaicism in Marfan and Ehlers-Danlos syndromes and related disorders. Eur J Hum Genet.

[40] Montgomery RA, Geraghty MT, Bull E (1998). Multiple molecular mechanisms underlying subdiagnostic variants of Marfan syndrome. Am J Hum Genet.

[41] Arnaud P, Morel H, Milleron O (2021). Unsuspected somatic mosaicism for FBN1 gene contributes to Marfan syndrome. Genet Med.

