# Supplementary material for: Causative role of a novel intronic indel variant in FBN1 and maternal germinal mosaicism in Marfan syndrome
Source: Orphanet J Rare Dis. 2024 May 21;19:209. doi: 10.1186/s13023-024-03139-4 (PMC11110283; doi:10.1186/s13023-024-03139-4)
Supplement: Supplementary file 1 — Supplementary Material 1. [file 13023_2024_3139_MOESM1_ESM.docx]

**SUPPLEMENTARY MATERIALS**

**Supplementary Figure 1. Screenshot of the exome alignment of c.3464-5_3464-4delGAinsAG in the proband (the black box).**


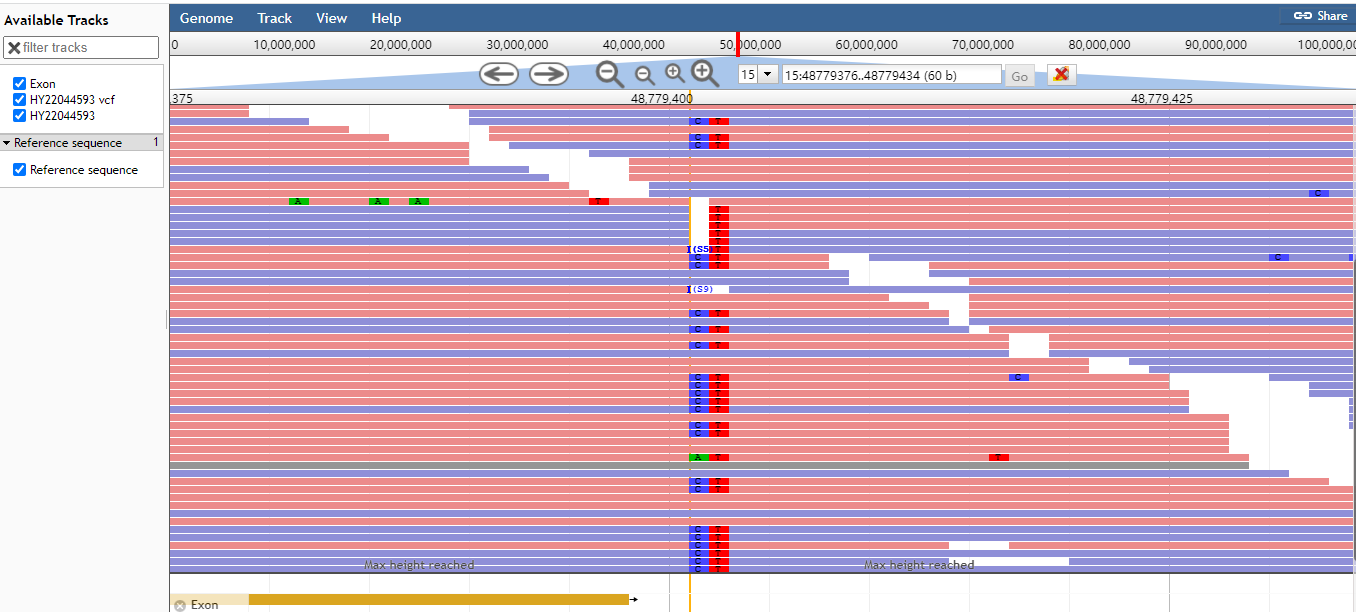


**Supplementary Figure 2. Screenshot of the exome alignment of c.3605_3606delGCinsTT p. (Ser1202Ile) in the proband (the black box).**


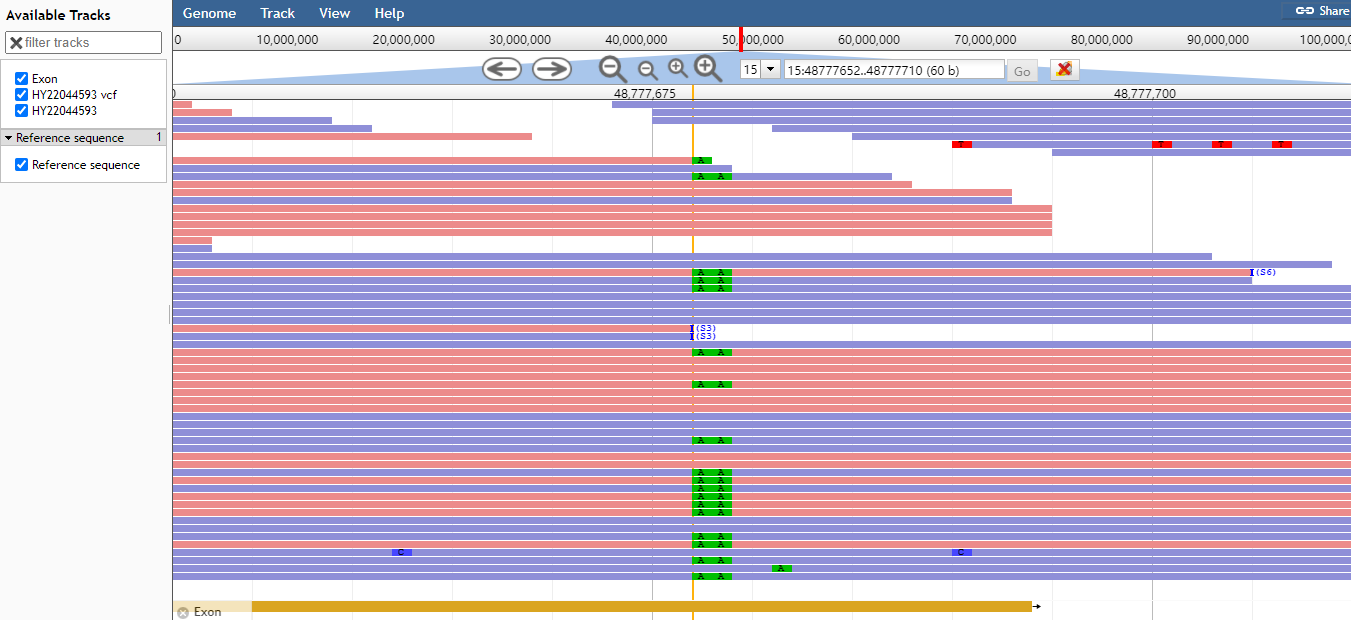


**Supplementary Figure 3.** **Results of Sanger sequencing confirmed that the heterozygous variant c.3605_3606delGCinsTT p. (Ser1202Ile) in the proband was inherited from the mother.**


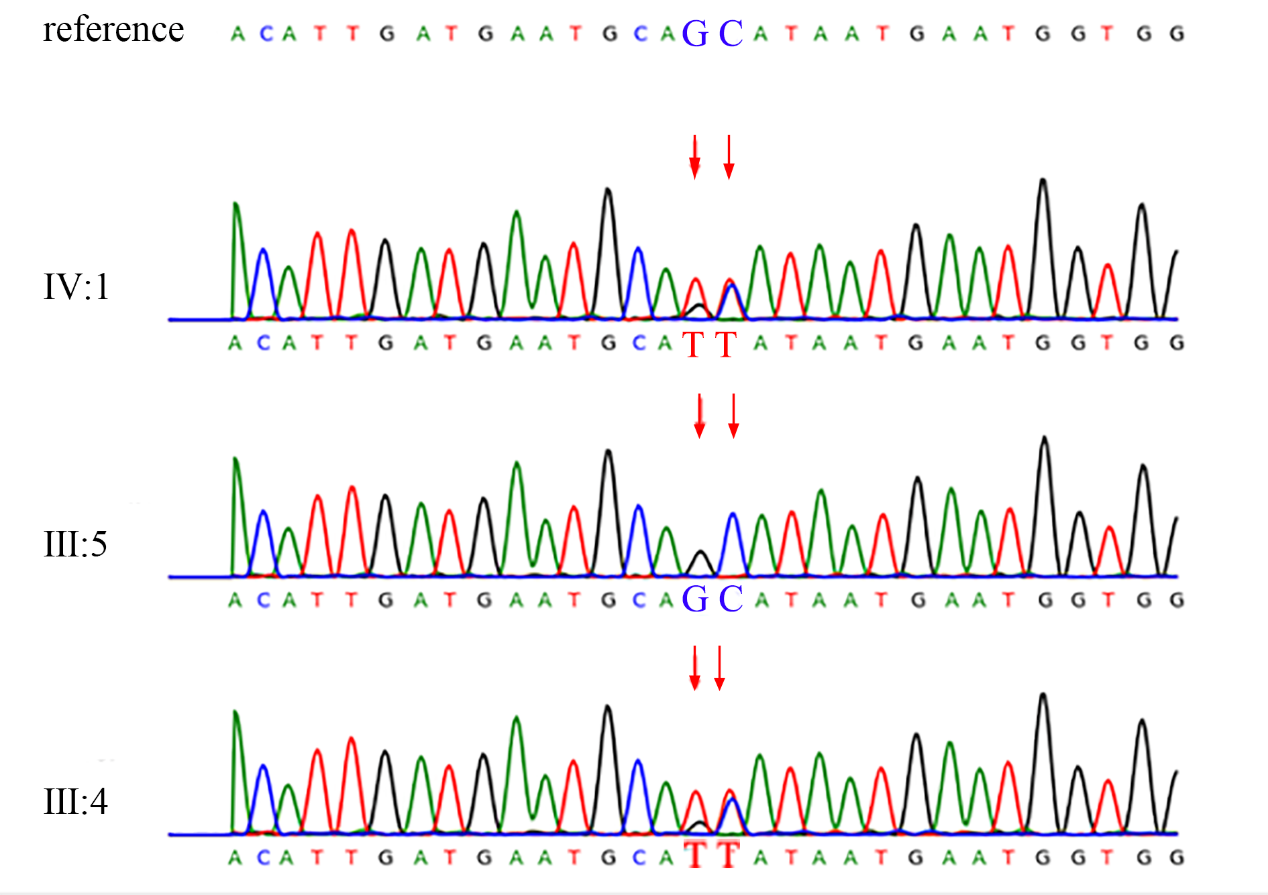


**Supplementary Figure 4.** **The Splicing damaging prediction of c.3464-5_3464-4delGAinsAG using Neural Network (BDGP).**

Wild type


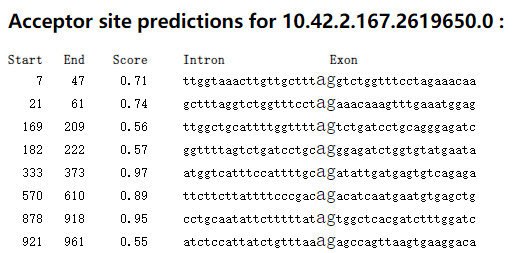


**

c.3464-4A>G


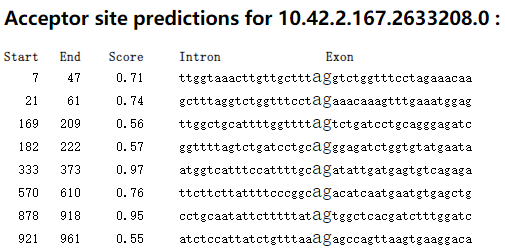


**

**c.3464-5_3464-4delGAinsAG**
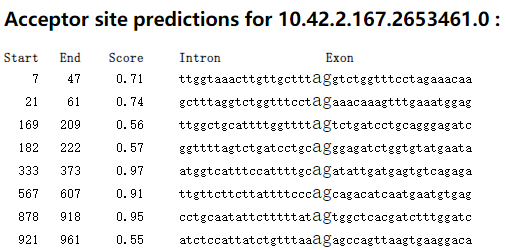


**

Supplementary Figure 4. The Splicing damaging prediction of c.3464-5_3464-4delGAinsAG using Neural Network (BDGP). The nucleic acid bases of c.3464-5 and c.3464-4 was marked with red star. Acceptor site predictions of c.3464-4A>G were the same as the wild type. Compared to the wild type, the variant c.3464-5_3464-4delGAinsAG resulted in generating the new acceptor site and insertion of three nucleic acid bases(cag). The insertion bases were shown with a red ellipse.

**Supplementary Figure 5. QF-PCR results of the family members (Ι:1, II:5).**


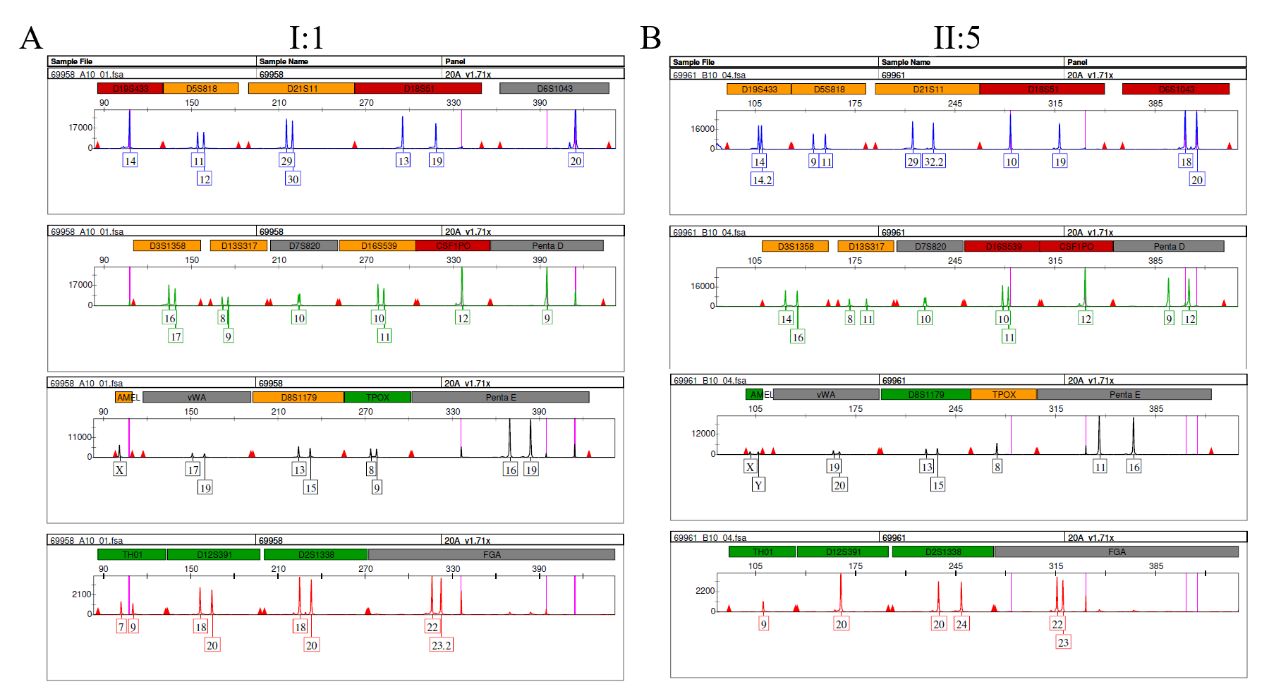


**Supplemental Figure 6. Germline mosaicism in family member I:1 by STR analysis.**


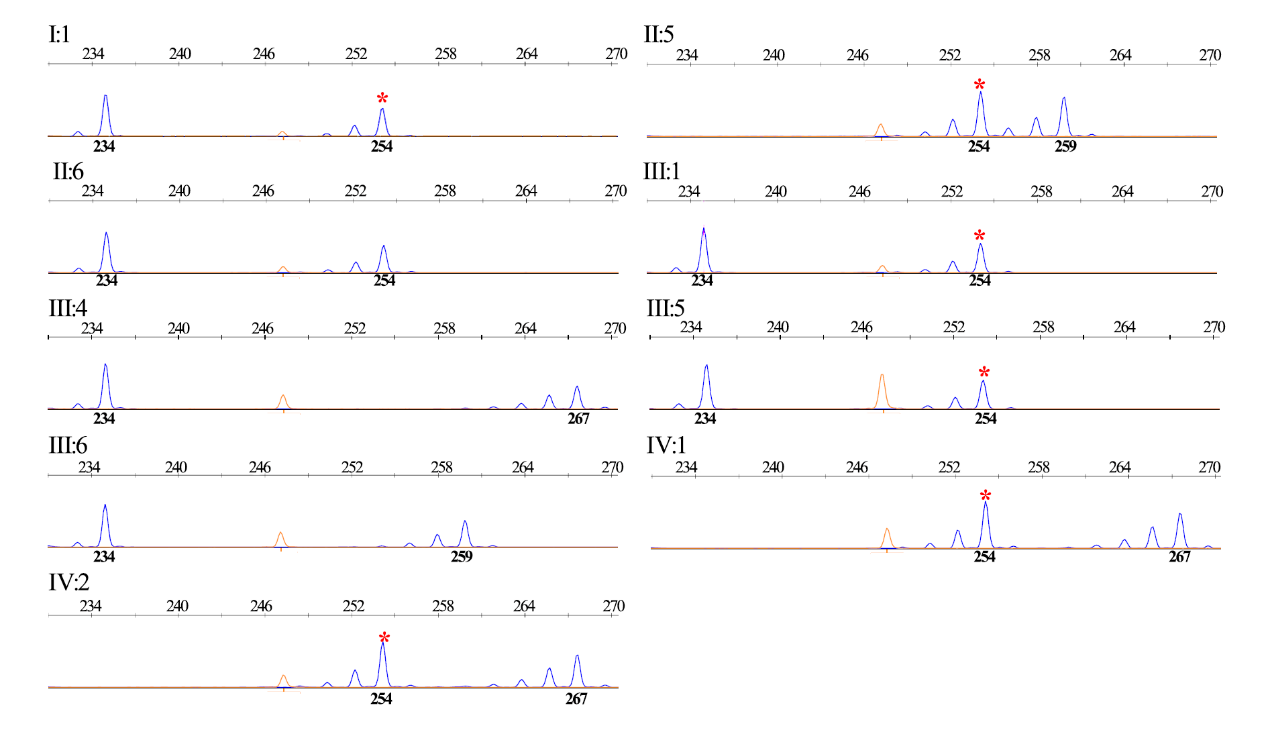


Supplemental Figure 6. Germline mosaicism in family member I:1 by STR analysis. Genetic mapping by typing highly polymorphic STS marker D15S992 established linkage to the *FBN1* gene in all the members excluding family member I:1. The mutant allele peak is marked as a red asterisk. I:1, with normal phenotype, but had mutant allele peak 254. Genotypic results together with two sons with MFS documented a mosaicism of FBN1 mutation in the blood cells of I:1, in accordance with Sanger sequences.

**Supplementary Figure 7. Agarose gel electrophoresis of RT‐PCR products in the whole blood(A) and HEK 293T(B).**


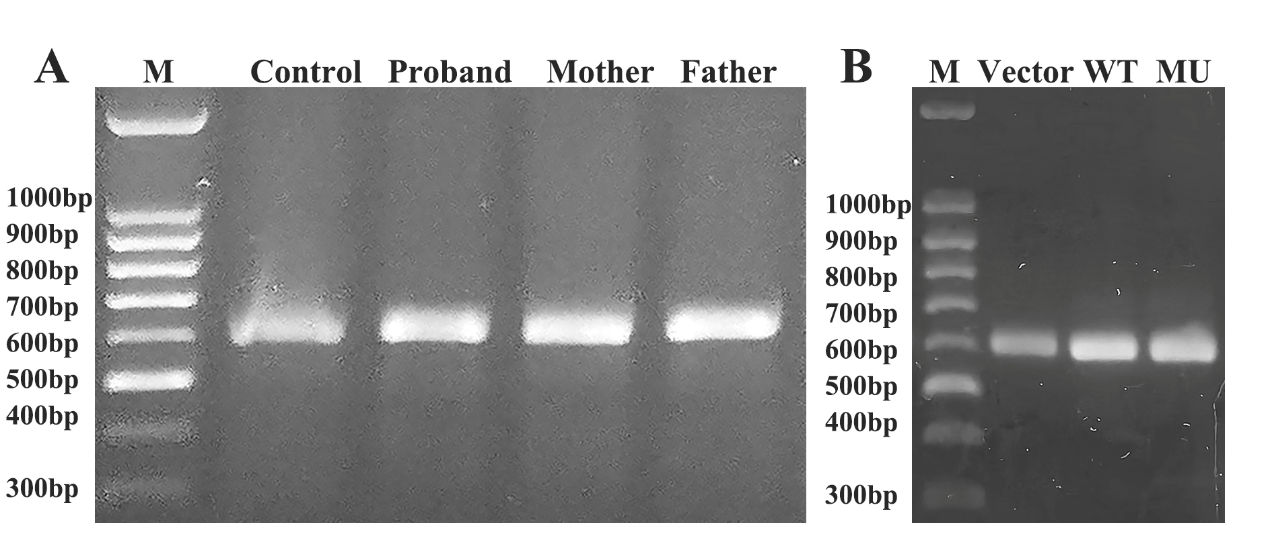
Supplementary Figure 7. Agarose gel electrophoresis of RT‐PCR products in the whole blood(A) and HEK 293T(B). M: marker, Vector: empty vector pCDNA3.1; WT: wild-type, MU: mutant-type.

**Supplementary Table 1. Primers for Sanger sequencing.**

| **Primer** | **Primer sequence (5’-3’)** | **PCR product** |
| --- | --- | --- |
| *FBN1*-E29F | AACATCTCCGCGTGTATCGG | 239 bp |
| *FBN1*- E29R | TCGGGAGTTGAATGGTAGCC |  |
| *FBN1*- E30F | GCCCTGCCTCTTAAATAGTGTT | 423 bp |
| *FBN1*- E30R | TATGCAGGCAATTTGAACTTC |  |
| D15S992*-*F | AGCTGAGAAATGCCTTCTATAAAT (5`6-FAM) | 228 bp~274 bp |
| D15S992-R | GAGGGCCACCTTGATAGT |  |
| p-IN25F | CACTGTGCTGGATATCTGCAGAATTCGGGCATTGAGACCTCCTGACT | 4679 bp |
| p-E31R | CTGATCAGCGGTTTAAACTTAAGCTTAGGGATATTTGTGCACTGACCA |  |
| RT-26F | GCGGCTTTGCTCTTGATTCT | 605 bp |
| RT-31R | AGGGATATTTGTGCACTGACCA |  |

**Supplementary Table 2. All variants in exons and splice region of *FBN1* gene.**

| **Gene** | **transcript** | **Exon** | **hgvs.c** | **hgvs.p** | **type** | **Genotype** | **MODE** | **dbsnp** | **ExAC_EAS** | **ref/alt** | **Level** |
| --- | --- | --- | --- | --- | --- | --- | --- | --- | --- | --- | --- |
| ***FBN1*** | **NM_000138.4** | **exon30** | **c.3605G>T** | **p.Ser1202Ile** | **Missense_variant** | **het** | **CIS** | **rs758542279** | **0.00012** | **29/23** | **VUS** |
| ***FBN1*** | **NM_000138.4** | **exon30** | **c.3606C>T** | **p.Ser1202Ser** | **synonymous_variant** | **het** | **CIS** | **rs750517637** | **0.00012** | **29/24** | **VUS** |
| ***FBN1*** | **NM_000138.4** | **intron28** | **c.3464-4A>G** | **-** | **splice_region_variant** | **het** | **CIS** | **rs1479967802** | **-** | **51/45** | **VUS** |
| *FBN1* | NM_000138.4 | intron28 | c.3464-5G>A | - | splice_region_variant | het | CIS | rs11853943 | 0.35220 | 52/47 | Benign |
| *FBN1* | NM_000138.4 | exon57 | c.6888G>A | p.Gln2296Gln | synonymous_variant | het | NA | rs363830 | 0.07964 | 50/50 | Benign |
| *FBN1* | NM_000138.4 | exon56 | c.6855T>C | p.Asp2285Asp | synonymous_variant | het | NA | rs363836 | 0.07967 | 38/29 | Benign |
| *FBN1* | NM_000138.4 | exon16 | c.1875T>C | p.Asn625Asn | synonymous_variant | het | NA | rs25458 | 0.35265 | 43/44 | Benign |

**Supplementary Table 3. All variants in exons and splice region of *FBN1* gene.**

| software | c.3464-4A>G | cut-off |
| --- | --- | --- |
| spliceAI | <0.2 | >0.5 |
| dbSNV_ADA | 0.001 | >0.8 |
| dbSNV_RF | 0.074 | >0.8 |
| mmsplice_delta_logit_psi | -0.197 | >1.5/<-1.5 |
| mmsplice_pathogenicity | 0.342 | >1.5/<-1.5 |
| Spidex-Zscore | 0.871 | >2/<-2 |

**Supplementary Table 4. ALL *FBN1* intronic indels variants affecting non-canonical splice sites included in Clinvar database.**

| Supplemental table 4. | | | | | |
| --- | --- | --- | --- | --- | --- |
| Gene | Variation | Clinical significance (Last reviewed) | Condition(s) | Accession | dbSNP ID |
| *FBN1* | c.6997+17_6997+18delinsGT | Likely benign(Last reviewed: Oct 19, 2021) | Familial thoracic aortic aneurysm and aortic dissection\|Marfan syndrome | VCV001582609 | - |
| *FBN1* | c.6380-12delinsTG | Likely benign(Last reviewed: Mar 11, 2019) | Familial thoracic aortic aneurysm and aortic dissection | VCV000927760 | rs2043078856 |
| *FBN1* | c.5066-13_5066-12delinsTAT | Uncertain significance(Last reviewed: Sep 15, 2019) | Familial thoracic aortic aneurysm and aortic dissection | VCV000924369 | rs2043294261 |
| *FBN1* | c.5066-13_5066-12delinsTAA | Likely benign(Last reviewed: Dec 31, 2018) | Familial thoracic aortic aneurysm and aortic dissection | VCV000922060 | rs2043294261 |
| ***FBN1*** | **c.3464-5_3464-3delinsAAT** | **Likely benign(Last reviewed: Nov 9, 2018)** | **Familial thoracic aortic aneurysm and aortic dissection** | VCV000922626 | rs2043515456 |
| ***FBN1*** | **c.3464-5_3464-4delinsAG** | **Uncertain significance(Last reviewed: Aug 19, 2019)** | **not provided** | VCV001302935 | - |
| *FBN1* | c.2420-9_2420-8delinsAGC | Uncertain significance(Last reviewed: Feb 11, 2016) | not provided | VCV000495576 | rs1555399217 |
| *FBN1* | c.2419+3delinsTTTTAGATCCATATTTTAG | Uncertain significance | Arthrogryposis, renal dysfunction, and cholestasis 1 | VCV001332758 | - |
| *FBN1* | c.989-10_989-8delinsATA | Likely benign(Last reviewed: Oct 8, 2018) | Familial thoracic aortic aneurysm and aortic dissection | VCV000927684 | rs2043848077 |
| *FBN1* | c.165-8_165-6delinsGG | Uncertain significance(Last reviewed: Apr 6, 2021) | Familial thoracic aortic aneurysm and aortic dissection\|not provided | VCV000919446 | rs2044669818 |
